# The Pioneer platform: A novel approach for selection of selective anti-cancer cytotoxic activity in bacteria through co-culturing with engineered human cells

**DOI:** 10.1371/journal.pone.0286741

**Published:** 2023-06-06

**Authors:** Gavin D. Garland, Kiran R. Patil, Suzanne D. Turner, Anne E. Willis

**Affiliations:** 1 MRC Toxicology Unit, University of Cambridge, Cambridge, United Kingdom; 2 Department of Pathology, University of Cambridge, Cambridge, United Kingdom; 3 CEITEC, Masaryk University, Brno, Czech Republic; Zagazig University Faculty of Veterinary Medicine, EGYPT

## Abstract

Most of the small-molecule drugs approved for the treatment of cancer over the past 40 years are based on natural compounds. Bacteria provide an extensive reservoir for the development of further anti-cancer therapeutics to meet the challenges posed by the diversity of these malignant diseases. While identifying cytotoxic compounds is often easy, achieving selective targeting of cancer cells is challenging. Here we describe a novel experimental approach (the Pioneer platform) for the identification and development of ‘pioneering’ bacterial variants that either show or are conduced to exhibit selective contact-independent anti-cancer cytotoxic activities. We engineered human cancer cells to secrete Colicin M that repress the growth of the bacterium *Escherichia coli*, while immortalised non-transformed cells were engineered to express Chloramphenicol Acetyltransferase capable of relieving the bacteriostatic effect of Chloramphenicol. Through co-culturing of *E*. *coli* with these two engineered human cell lines, we show bacterial outgrowth of DH5α *E*. *coli* is constrained by the combination of negative and positive selection pressures. This result supports the potential for this approach to screen or adaptively evolve ‘pioneering’ bacterial variants that can selectively eliminate the cancer cell population. Overall, the Pioneer platform demonstrates potential utility for drug discovery through multi-partner experimental evolution.

## Introduction

Cancer is a major public health concern globally and a leading cause of death [1, 2]. There is an urgent and ongoing need to discover and develop new therapeutic agents to better target malignant diseases with improved efficacy and alternative pharmacological activities to exploit different disease vulnerabilities. Remarkably, most of the small-molecule drugs approved for the treatment of cancer over the past 40 years are natural compounds and their derivatives or analogues [3, 4]. Indeed, bacterial sources have proven to be an important reservoir for drug discovery providing 3,800 known bioactive natural compounds with many more likely yet to be discovered [4, 5]. Previous studies have used cell lysates and supernatants from bacteria engineered to express and secrete toxins to screen for anti-cancer activity against a cancer cell line monolayer [6]. More recently, a bacteria-in-spheroid co-culture (BSCC) platform has been utilised to screen a selection of therapeutic payloads including bacterial toxins and anti-cancer peptides in the context of tumour spheroids [7]. But such approaches require pre-knowledge of bioactive agents to be tested and does not account for off-target toxicity which can limit treatment efficacy. Therefore, there would be an obvious utility for a novel platform by which bacterial variants that display selective contact-independent anti-cancer activity could be identified and the responsible bioactive agents optimised.

Colicin M is a plasmid-encoded toxin which is produced and released into the extracellular environment by certain strains of *Escherichia coli* (*E*. *coli*) to kill competitors of the same or related species [8, 9]. Its uptake requires specific binding to the FhuA outer membrane receptor and subsequent energy-dependent translocation into the periplasm by the TonB machinery [9–11]. Once internalized, Colicin M exerts bactericidal activity by interfering with murein biosynthesis to provoke cell lysis [8, 9, 12]. Colicin M shows a narrow antibacterial spectrum owing to the specificity of its import system [9]. This, combined with the fact that eukaryotic cells lack a peptidoglycan cell wall, means that Colicin M is not likely to exert any cytotoxic effect against human cells.

Chloramphenicol is a bacteriostatic antibiotic which is effective against most strains of *E*. *coli* [13]. It inhibits protein synthesis, binding the 23S rRNA of the 50S ribosomal subunit to inhibit the peptidyl transferase activity of the bacterial ribosome [14–16]. In some strains of *E*. *coli*, resistance to Chloramphenicol has developed through the expression of Chloramphenicol Acetyltransferase (CAT). CAT acetylates Chloramphenicol to detoxify it, preventing Chloramphenicol binding to the ribosome [17]. While Chloramphenicol does not bind the eukaryotic cytoplasmic ribosome, it can associate with mammalian mitochondrial ribosomes and interfere with mitochondrial translation [16].

Hypothetically, cancer cells engineered to impose a negative selection pressure through Colicin M and non-transformed cells engineered to enforce a positive selection pressure through CAT could be used to constrain the outgrowth of co-cultured *E*. *coli* unless the bacteria could selectively eliminate the source of the negative selection pressure. In this study, we develop this hypothesis into an experimental platform (referred to as the Pioneer platform) and describe the resulting screen for the identification and development of bacterial variants that either show or are conduced to exhibit selective contact-independent anti-cancer cytotoxic activities.

## Materials and methods

### Constructs and cloning

Double-stranded DNA fragments encoding the Colicin M activity gene (*cma*) from *E*. *coli* bearing a C-terminal His_6_-tag (as per the sequence of the gene in the pMLD190 plasmid [12]) and *cat* from *E*. *coli* (as per the sequence of the gene in the JR66b plasmid [18]) codon-optimised for *Homo sapiens* were synthesised (Thermofisher Scientific) (S1 Table) and amplified by PCR using pLVX c1F/R primers. The PCR products were digested with NotI and XhoI restriction enzymes and ligated with NotI/XhoI-digested pLVX-IRES-ZsGreen1 and pLVX-IRES-mCherry plasmids (Takara Bio), respectively. The resulting pLVX-Cma-ZsG1 and pLVX-Cat-mCh plasmids were verified by Sanger sequencing.

Complementary single-stranded DNA fragments encoding the signal peptide sequence from the human *IL-2* gene (as described in [19]) codon-optimised for *Homo sapiens* (IL-2 SP Oligo F/R) were synthesized (Sigma-Aldrich) (S1 Table) and annealed. The annealed product was ligated with EcoRI/SpeI-digested pLVX-Cma-ZsGreen1 plasmid. The resulting pLVX-SP-Cma-ZsG1 plasmid was verified by Sanger sequencing.

The N125G mutation of *cma* was introduced using the QuikChange Lightning Site-Directed Mutagenesis Kit (Stratagene) using N125G QCL F and R primers (S1 Table) and pLVX-SP-Cma-ZsG1 plasmid as a template. The mutation in the resulting pLVX-SP-Cma(N125G)-ZsG1 plasmid was verified by Sanger sequencing.

### Human cell culture

293FT cells (obtained from Thermo Fisher Scientific, R70007), DLD-1 cells (a kind gift from Dr. Douglas Winton, University of Cambridge, UK) [20] and DLD-1 Cma-ZsG1 cells were cultured in DMEM (Gibco 41966) supplemented with 10% FCS at 37°C/5% CO_2_. HCEC 1CT cells (a kind gift from Prof. Jerry Shay, UT Southwestern Medical Centre, USA) [21] and HCEC 1CT Cat-mCh cells were cultured in Medium X (DMEM supplemented with 2% FCS, 2μg/ml Apo-Transferrin, 10 μg/ml Human Insulin, 20 ng/ml Epidermal Growth Factor, 5 nM Sodium Selenite and 1 μg/ml Hydrocortisone) at 37°C in 5% CO_2_/5% O_2_ as previously described [21].

### Generation of cell lines

For lentivirus production, 293FT cells were seeded 24 hours in advance of transfection at a density of 0.5 × 10^6^ cells per well of a six-well plate. 4 μg psPAX2, pMD2.G and pLVX-SP-Cma(N125G)-ZsG1 or pLVX-Cat-mCh lentiviral DNA were co-transfected at a 1:1:1 molecular ratio with 6 μl Turbofect transfection reagent (Thermo Fisher Scientific). Lentiviral supernatant was collected 48 hours and 72 hours later, and pooled after filtering through a 0.45-μm cellulose-acetate filter.

The colorectal adenocarcinoma (DLD-1) and immortalised human colonic epithelial (HCEC 1CT) cell lines were seeded 24 hours before transduction to reach 20–30% confluency before filtered lentiviral supernatant was added with 8 or 4 μg/ml Polybrene, respectively, and cells collected by centrifugation at 1200 xg for 1 hour at 32°C. ZsGreen1- and mCherry-fluorescent cell populations were isolated by FACS on a FACS Aria Fusion flow cytometer (BD).

### Flow cytometry

Trypsinised cells were washed with PBS and fixed in 2% paraformaldehyde/PBS at 4°C overnight. Fixed cells were washed in PBS and resuspended in flow cytometry buffer (1% Bovine Serum Album/PBS). ZsGreen1 and mCherry fluorescence was analysed on a FACS Fortessa I cytometer (BD). 10,000 events were recorded and data analysed with FlowJo v9.1 (BD).

### SDS–PAGE and western blot analysis

Cell extracts and conditioned media samples were denatured in 1× SDS–PAGE Loading Buffer (50 mM Tris 6.8, 2% SDS, 0.1% Bromophenol Blue, 10% glycerol, 25 mM DTT) at 95°C for 5 min. Denatured samples were separated on 12% SDS-PAGE gels and proteins transferred to Immobilon polyvinylidene fluoride (PVDF) membrane. Membranes were blocked in 5% BSA and incubated with primary antibodies overnight at 4°C. Primary antibodies were as follows: monoclonal mouse anti-poly-His (Sigma-Aldrich H1029; diluted 1:5000), polyclonal rabbit anti-CAT (Sigma-Aldrich C9336; diluted 1:2000) and monoclonal mouse anti-TUBA (Sigma-Aldrich T9026; diluted 1:10 000). Membranes were then incubated with secondary antibodies for 1 h at room temperature. Secondary antibodies were as follows and diluted 1:10,000: anti-mouse HRP-immunoglobulins (Dako P0260) and anti-rabbit HRP-immunoglobulins (Dako P0448). Membranes were developed with Immobilon Western Chemiluminescent HRP Substrate (Merck Millipore) and imaged with an ImageQuant LAS-4000 imaging system (GE Life Sciences).

### Soft agar colony formation assays

Soft agar colony formation assays were performed as previously described [22]. Briefly, 5 × 10^3^ DLD-1 and HCEC-1CT cells were resuspended in 0.3% agar/Medium X overlain above a layer of 0.6% agar/culture medium in a six-well plate and incubated at 37°C in 5% CO_2_/5% O_2_ for 14 days. Cells were stained with Nitro-Tetrazolium Blue Chloride solution after overnight incubation at 37°C in 5% CO_2_/5% O_2_.

### Conditioned media production and bacterial growth assays

DLD-1 and DLD-1 Cma-ZsG1 cells were seeded 24 hours in advance of harvest at a density of 1 × 10^6^ cells per well of a six-well plate. HCEC 1CT and HCEC 1CT Cat-mCh cells were seeded 24 hours in advance of harvest at a density of 0.5 × 10^6^ cells per well of a six-well plate with or without 6.8 μg/ml Chloramphenicol. Conditioned media was collected and filtered through a 0.45-μm cellulose-acetate filter.

For bacterial growth assays, starter cultures of DH5α *E*. *coli* (Thermo Fisher Scientific) were established in LB broth overnight at 37°C with shaking at 250 rpm. 1 ml 50% conditioned media/50% LB broth was inoculated with 10 μl bacterial culture and incubated at 37°C with shaking at 250 rpm for up to 8 hours. Bacterial growth was measured by absorbance at 600 nm (OD600) at the stated time points and data are presented relative to 8-hour non-conditioned media samples.

### Human-bacterial co-culture assays

DLD-1 and DLD-1 Cma-ZsG1 cells were seeded 24 hours in advance at a density of 0.5 × 10^6^ cells per well of a six-well plate. Culture media was replaced with 0.3% agar/DMEM with 10% FCS medium inoculated with 0.15 μl 10^−4^ DH5α *E*. *coli* starter culture dilution and incubated at 37°C in 5% CO_2_ for 3 days upside down.

HCEC 1CT and HCEC 1CT Cat-mCh cells were seeded 24 hours in advance at a density of 0.25 × 10^6^ cells per well of a six-well plate. Culture media was replaced with 0.3% agar/Medium X inoculated with 0.15 μl 10^−4^ DH5α *E*. *coli* starter culture dilution and incubated at 37°C in 5% CO_2_/5% O_2_ for 2 days upside down.

### Pioneer platform

DLD-1 Cma-ZsG1 and HCEC 1CT Cat-mCh cells were seeded at a density of 20 × 10^3^ and 10 × 10^3^ cells per well, respectively, in a 96-well plate in Medium X 24 hours before the assay was conducted. Growth media was replaced with 100 μl 0.3% agar/Medium X supplemented with 3.4 μg/ml Chloramphenicol, and inoculated with 0.01 μl 10^−4^ DH5α *E*. *coli* starter culture dilution before incubation at 37°C in 5% CO_2_/5% O_2_ for up to 3 days upside down. ZsGreen1 and mCherry fluorescence was analysed on a Spectramax i3 plate-reader at the stated time points and presented with background subtracted.

### Statistical analysis

Statistical tests were performed using GraphPad Prism 9 Software.

## Results

### The Pioneer platform concept

**Fig 1:**
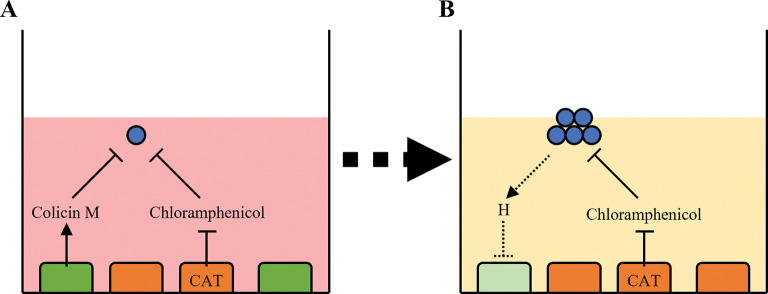
Principle of the Pioneer platform. In the Pioneer platform, *E*. *coli* bacteria (blue) are co-cultured in soft agar containing Chloramphenicol over a mixed monolayer of human cancer cells which are labelled with ZsGreen1 fluorescence and secrete Colicin M (green), and human immortalised non-transformed cells which are labelled with mCherry fluorescence and express CAT (red) which detoxifies Chloramphenicol. Bacterial growth is repressed by Colicin M so that there is no outgrowth of bacterial colonies and culture media is not discoloured (A). However, ‘pioneering’ bacteria which exhibit or can adapt to acquire contact-independent selective cytotoxic activity against the cancer cells (represented by dashed lines and hypothetical bioactive compound H) can eliminate the source of Colicin M without affecting the production of CAT from the immortalised non-transformed cells which relieves the negative selection pressure imposed by Chloramphenicol, and bacterial outgrowth can occur leading to a detectable colour change of the culture media (B).

In the Pioneer platform, *E*. *coli* are suspended in semi-solid agar/media, supplemented with Chloramphenicol to represses bacterial outgrowth, above a mixed monolayer of adherent human cells (Fig 1A). The mixed monolayer of adherent human cells consists of two engineered cell types: First, immortalised non-transformed cells engineered to co-express CAT enzyme and mCherry fluorescent protein. The CAT enzyme detoxifies Chloramphenicol in the local environment which enables sensitive *E*. *coli* to grow. Therefore, the bacteria are under positive selection pressure to maintain the survival of the CAT-expressing immortalised cells. Second, cancer cells engineered to express and secrete Colicin M while co-expressing ZsGreen1 fluorescent protein. Secretion of Colicin M into the local environment is bactericidal against susceptible *E*. *coli* and prevents their growth. Thus, the bacteria are under negative selection pressure to eliminate the Colicin M-producing cancer cells.

Under these conditions, bacterial outgrowth can be achieved by ‘pioneering’ bacteria which produce compounds that exhibit selective cytotoxic activity against the cancer cells while not affecting the growth of the immortalised non-transformed cells (Fig 1B). This would eliminate the local source of Colicin M (i.e., the cancer cells) while maintaining the source of CAT enzyme required to detoxify Chloramphenicol (i.e., the immortalised non-transformed cells). Consequently, expression of ZsGreen1 fluorescent protein expressed by the cancer cells would be depleted while expression of mCherry fluorescent protein expressed by the immortalised non-transformed cells would be unaffected. These ‘pioneering’ bacteria can then be isolated and expanded, and the mechanisms through which they exhibit selective anti-cancer activity can be validated, characterised and exploited for drug discovery.

### Generation of Colicin M secreting cells

In order to enforce a negative selection pressure against DH5α *E*. *coli*, the transformed human colon adenocarcinoma cell line DLD-1 was transduced with a pLVX-IRES-ZsGreen1 lentiviral vector in which a C-terminal poly-His tagged Colicin M (*Cma*) coding sequence optimised for human codon usage was cloned upstream of the IRES sequence (pLVX-Cma-ZsG1) [20]. To facilitate active secretion from DLD-1 cells, an IL2 Signal Peptide sequence was cloned into pLVX-Cma-ZsG1 upstream of the *Cma* transcription start site [19], and an N125G mutation was included in the *Cma* coding sequence to prevent glycosylation of this residue and to enable Colicin M to be actively secreted while retaining colicinogenic activity (pLVS-SP-Cma(N125G)-ZsG1). The transduced cells (DLD-1 Cma-ZsG1) also co-express ZsGreen1 fluorescent protein facilitating purification of transduced cells by fluorescence-activated cell sorting (FACS).

**Fig 2:**
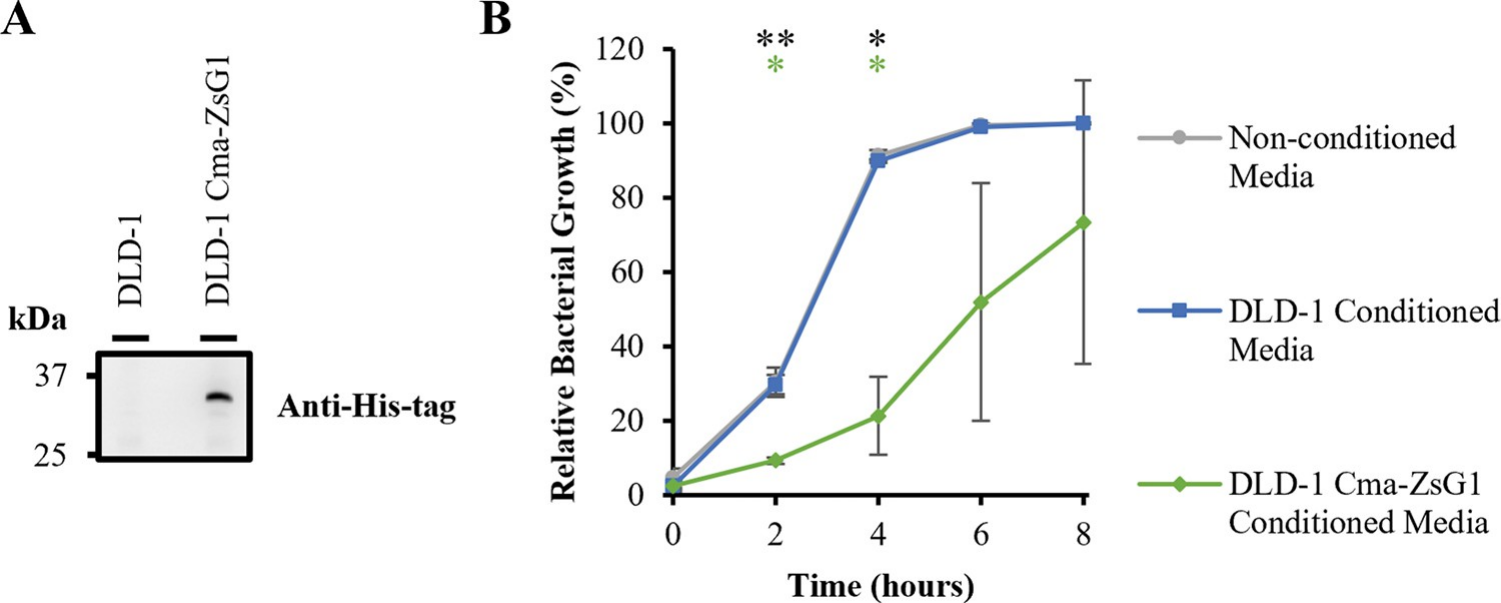
Detection and colicinogenic activity of His-tagged Colicin M in conditioned media from transduced DLD-1 Cma-ZsG1 cells. (A) Proteins in conditioned media from DLD-1 and DLD-1 Cma-ZsG1 cells cultured for 24 hours were separated by SDS–PAGE and visualised by Western blot with the indicated antibody. (B) Relative bacterial growth of DH5α *E*. *coli* grown in liquid culture in the presence of 50% non-conditioned media (grey circles) or conditioned media from DLD-1 (blue squares) or DLD-1 Cma-ZsG1 cells (green diamonds). Error bars represent means ± standard deviations; n = 3 independent experiments; * *P* < 0.05 and ** *P* < 0.01 by two-way ANOVA Tukey’s Multiple Comparisons Test compared with the non-conditioned media control (coloured asterisks) or comparing DLD-1 and DLD-1 Cma-ZsG1 samples (black asterisks).

His-tagged Colicin M is detectable in conditioned media from transduced DLD-1 Cma-ZsG1 cell cultures but not from non-transduced DLD-1 cells as demonstrated by Western blot (Fig 2A). In addition, co-expression of ZsGreen1 in transduced cells is detectable by flow cytometry (S1 Fig). Moreover, growth of DH5α *E*. *coli* is significantly repressed (P<0.05) when grown in the presence of 50% conditioned media isolated from transduced DLD-1 Cma-ZsG1 cell cultures compared to conditioned media from non-transduced DLD-1 cells or non-conditioned media (Fig 2B). These data confirm that the DLD-1 Cma-ZsG1 cells can exert a contact-independent negative selection pressure on the growth of DH5α *E*. *coli* through the colicinogenic activity of Colicin M actively secreted into conditioned media.

### Generation of CAT expressing cells

**Fig 3:**
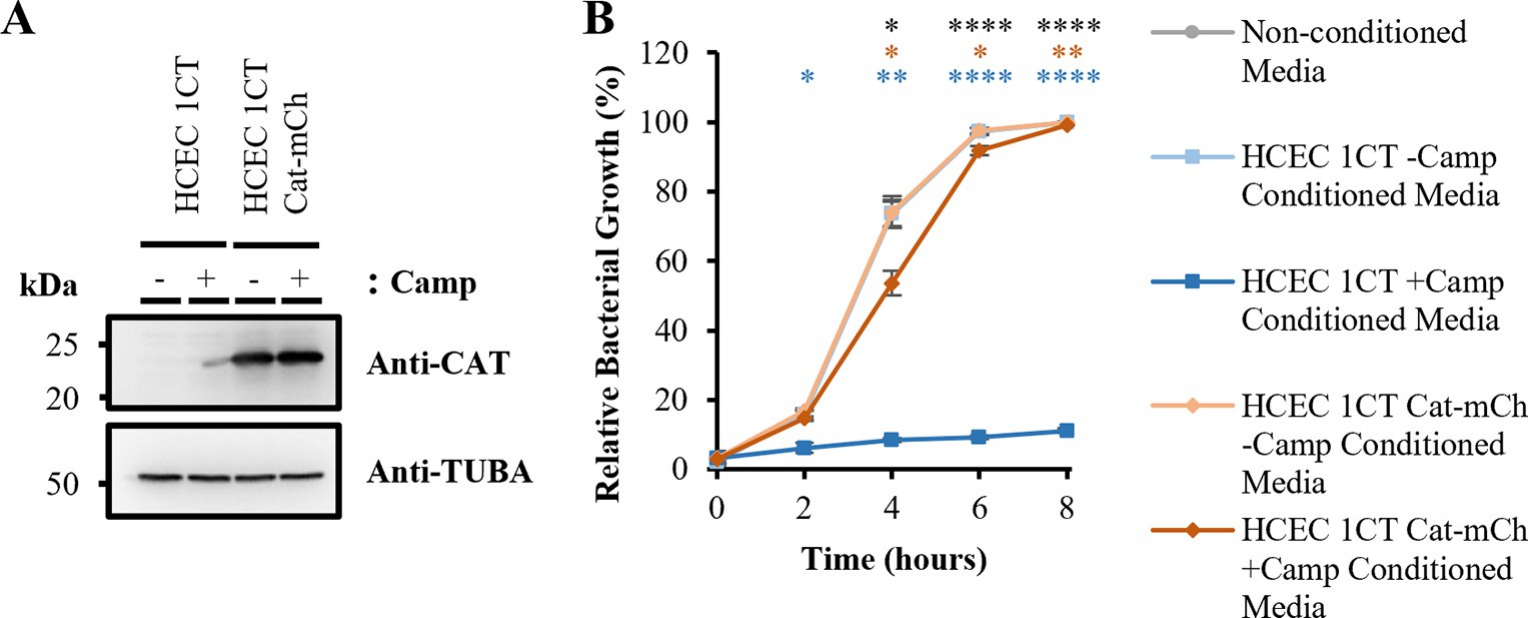
Expression of CAT in HCEC 1CT Cat-mCh cell lysates and detoxification of Chloramphenicol in conditioned media from transduced HCEC 1CT Cat-mCh cells. (A) Proteins from cell extracts of HCEC 1CT and HCEC 1CT Cat-mCh cells cultured with (+Camp) or without (-Camp) 6.8 μg/ml Chloramphenicol for 24 hours were separated by SDS–PAGE and visualised by Western blot with the indicated antibody. (B) Relative bacterial growth of DH5α *E*. *coli* grown in liquid culture in the presence of 50% non-conditioned media (grey circles) or conditioned media from HCEC 1CT (blue squares) and HCEC 1CT Cat-mCh (orange diamonds) cells -/+Camp. Error bars represent means ± standard deviations; *n* = 3 independent experiments; * *P* < 0.05, ** *P* < 0.01 and **** *P* < 0.0001 by two-way ANOVA Tukey’s Multiple Comparisons Test compared with non-conditioned media control (coloured asterisks) or comparing HCEC 1CT +Camp and HCEC 1CT Cat-mCh +Camp samples (black asterisks).

To enforce a negative selection pressure against DH5α *E*. *coli*, HCEC 1CT cells were employed. This non-transformed cell line has been immortalised through ectopic expression of hTERT and CDK4 [21], and are unable to form colonies in soft agar unlike the transformed DLD-1 cell line (S2 Fig). HCEC 1CT cells were transduced with a pLVX-IRES-mCherry lentiviral vector in which a Chloramphenicol Acetyltransferase gene (*Cat*) coding sequence optimised for human codon usage was cloned upstream of the IRES sequence (pLVX-Cat-mCh) leading to CAT expression which is not affected by Chloramphenicol (Fig 3A). The transduced cells (HCEC 1CT Cat-mCh) also co-express mCherry fluorescent protein facilitating their purification by FACS (S3 Fig).

Interestingly, growth of DH5α *E*. *coli* is significantly repressed (P<0.0001) when grown in the presence of 50% conditioned media from non-transduced HCEC 1CT cells cultured with 6.8 μg/ml Chloramphenicol compared to non-conditioned media or conditioned media not supplemented with Chloramphenicol (Fig 3B). In contrast, DH5α *E*. *coli* grown in the presence of 50% conditioned media from transduced HCEC 1CT Cat-mCh cells cultured with 6.8 μg/ml Chloramphenicol is significantly higher (P<0.001) and is almost restored to the level of growth seen when Chloramphenicol is absent from conditioned media. Moreover, the presence of HCEC 1CT Cat-mCh cells (to condition the media) does not affect the growth of DH5α *E*. *coli* in the absence of Chloramphenicol (Fig 3B). Therefore, the HCEC 1CT Cat-mCh cells are able to exert a contact-independent positive selection pressure on the growth of DH5α *E*. *coli* through the detoxification of Chloramphenicol in conditioned media.

### Human-bacterial cell co-culture

To facilitate molecular interactions between bacterial and human cells so that the bacteria can exert contact-independent selective pressure upon the human cells, it is necessary to co-culture bacterial cells with human cells under semi-solid culture conditions.

**Fig 4:**
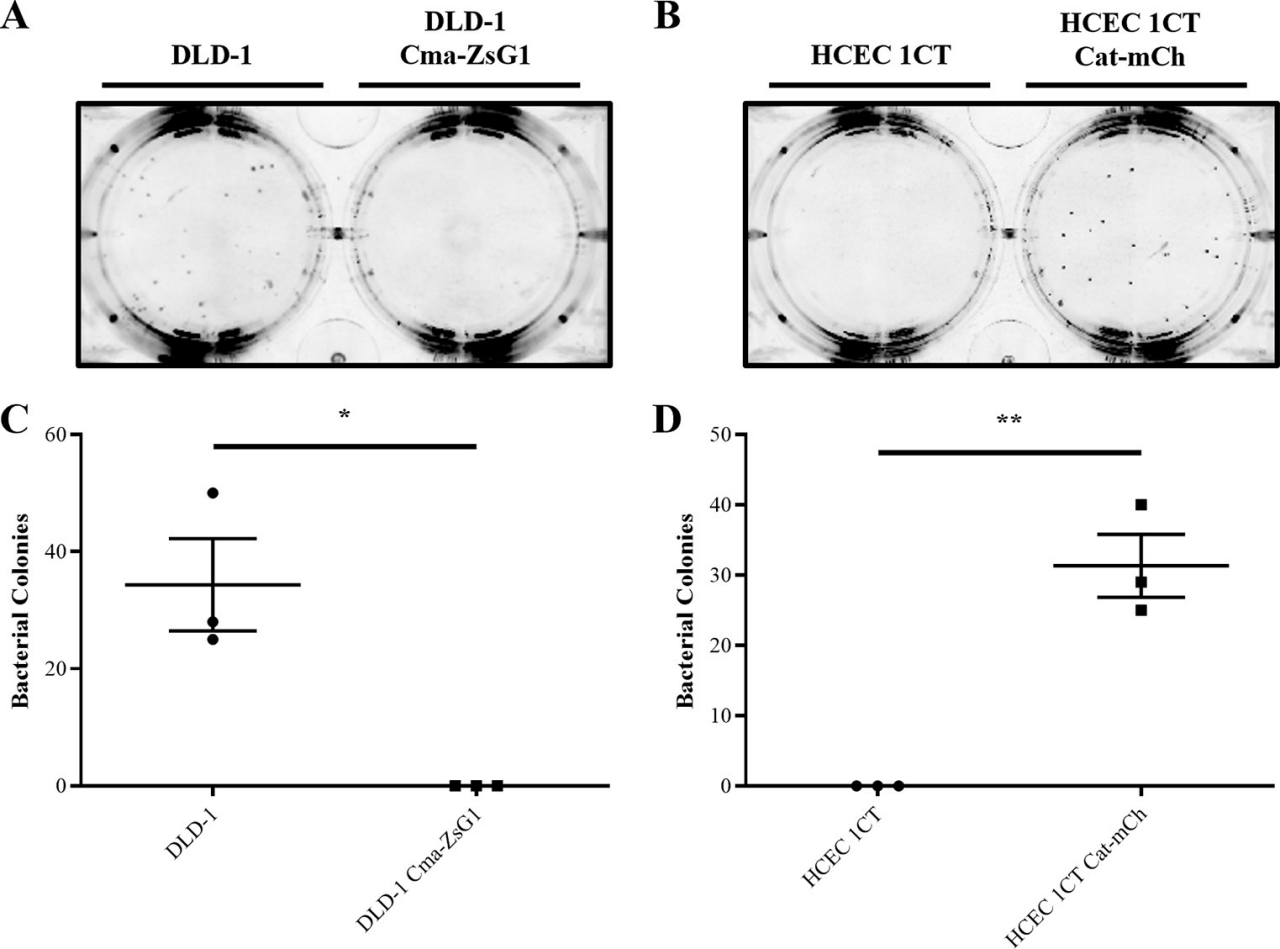
Co-culture of *E*. *coli* with DLD-1 Cma-ZsG1 cells represses bacterial growth but co-culture with HCEC 1CT Cat-mCh cells promotes bacterial growth in the presence of Chloramphenicol. Growth of DH5α *E*. *coli* colonies co-cultured in soft agar above a monolayer of human DLD-1 and DLD-1 Cma-ZsG1 cells for 3 days (A and C) or with 3.4 μg/ml Chloramphenicol above a monolayer of human HCEC 1CT or HCEC 1CT Cat-mCh cells for 2 days (B and D). Representative images from independent replicative experiments are shown (A and B). Scatter plots show error bars representing means ± standard deviations; *n* = 3 independent experiments; * *P* < 0.05 and ** *P* < 0.01 by unpaired student’s t-test assuming unequal variances (C and D).

To achieve this, an adherent monolayer of non-transduced DLD-1 or transduced DLD-1 Cma-ZsG1 human cells was established and then overlain with a layer of soft agar inoculated with DH5α *E*. *coli*. After 3 days of co-culture, there was significant outgrowth (P<0.05) of bacterial colonies in the presence of the non-transduced DLD-1 cell monolayer, whereas no bacterial outgrowth was observed in the presence of the transduced DLD-1 Cma-ZsG1 cell monolayer (Fig 4A and 4C). These data confirm that the DLD-1 Cma-ZsG1 cell monolayer secretes Colicin M which exerts a colicinogenic effect in a semi-solid culture environment, and thus enforces a significant negative selection pressure against DH5α *E*. *coli*.

Similarly, an adherent monolayer of non-transduced HCEC 1CT or transduced HCEC 1CT Cat-mCh human cells was established and then overlain with a layer of soft agar containing 3.4 μg/ml Chloramphenicol inoculated with DH5α *E*. *coli*. After 2 days of co-culture, there was no outgrowth of bacteria in the presence of the non-transduced HCEC 1CT cell monolayer, whereas there was significant outgrowth (P<0.01) of bacterial colonies in the presence of the transduced HCEC 1CT Cat-mCh cell monolayer (Fig 4B and 4D). Therefore, these data show that the HCEC 1CT Cat-mCh cell monolayer can detoxify Chloramphenicol through the activity of exogenously expressed CAT enzyme to promote the growth of DH5α *E*. *coli* in a semi-solid culture environment and thus exerts a significant positive selection pressure.

### The Pioneer platform in practice

To assess whether DH5α *E*. *coli* are capable of overcoming the negative selection pressure imposed by DLD-1 Cma-ZsG1 cells without jeopardising the positive selection pressure provided by HCEC 1CT Cat-mCh cells, and cause bacterial outgrowth under the conditions of the Pioneer platform, an adherent monolayer of DLD-1 Cma-ZsG1 cells, HCEC 1CT Cat-mCh cells or a mixture of both was established and then overlain with a layer of soft agar containing 3.4 μg/ml Chloramphenicol inoculated with DH5α *E*. *coli*.

**Fig 5:**
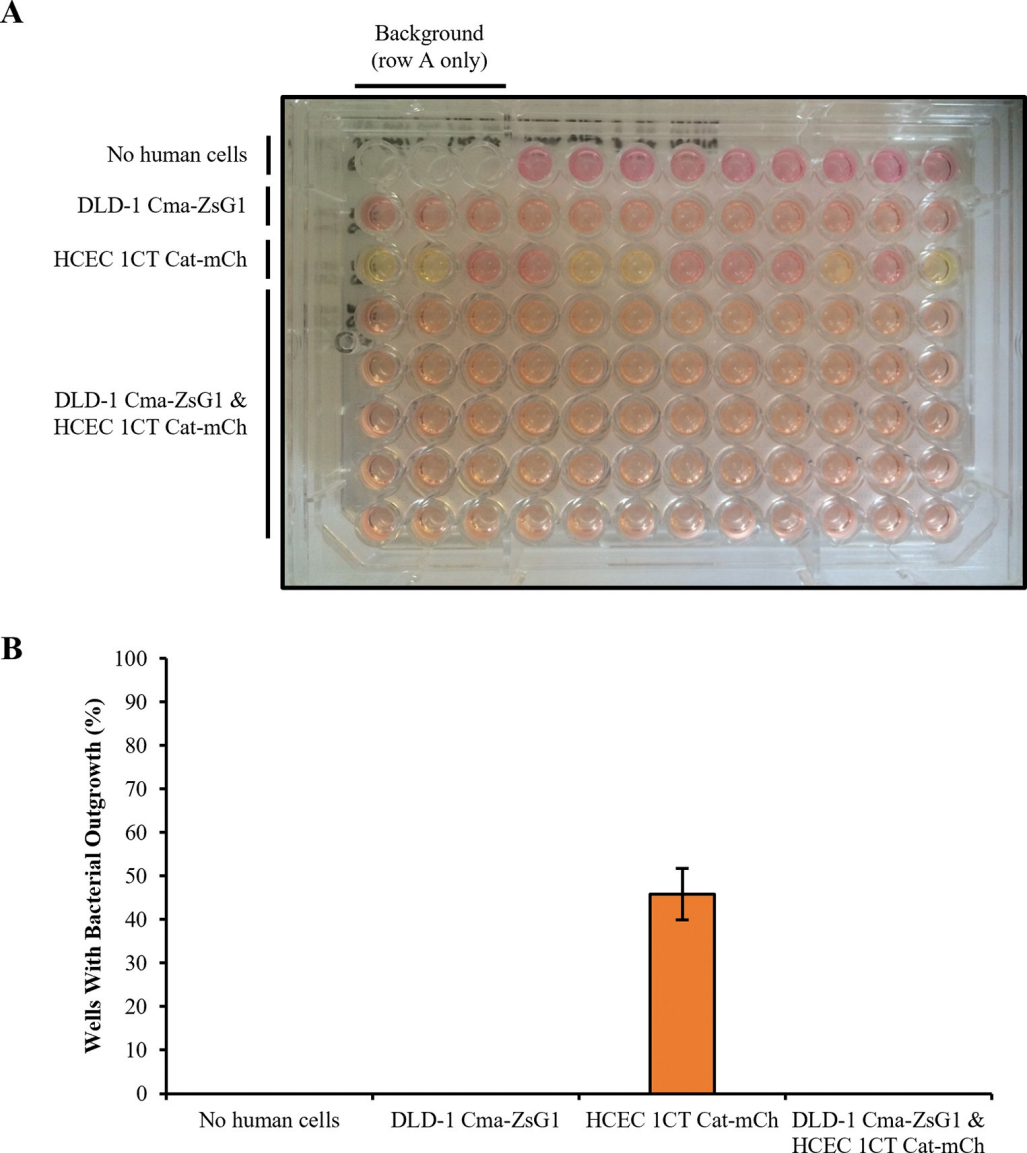
Bacterial outgrowth is repressed by a mixed monolayer of DLD-1 Cma-ZsG1 and HCEC 1CT Cat-mCh cells in the Pioneer platform. Growth of DH5α *E*. *coli* bacterial colonies co-cultured in soft agar with 3.4 μg/ml Chloramphenicol without human cells (row A), or above a monolayer of DLD-1 Cma-ZsG1 cells (row B) or HCEC 1CT Cat-mCh cells (row C) or a mixed monolayer of both (rows D-H) for 3 days. Empty wells (A1-A3) were used for background subtraction. A representative image from independent replicative experiments showing culture media discolouration and bacterial colony formation is shown (A), and data are represented in a bar chart showing error bars representing means ± standard deviations (B); *n* = 2 independent experiments.

The results show that in the absence of the HCEC 1CT Cat-mCh cell monolayer, none of the wells show outgrowth of bacterial colonies or discolouration of culture media which remains pink (Fig 5A (rows A and B) and 5B). This confirms that the negative selection pressure caused by Chloramphenicol is sufficient to prevent bacterial outgrowth. In contrast, in the presence of the HCEC 1CT Cat-mCh cell monolayer only, 6 out of 12 wells show discolouration of culture media to yellow, which is caused by outgrowth of 1–2 bacterial colonies in 5 wells (C1, C2, C5, C6 and C12) and diffuse bacterial outgrowth in another well (C10) (Fig 5A (row C) and 5B). This suggests that in approximately half of the wells, the positive selection pressure provided by the HCEC 1CT Cat-mCh cell monolayer through the expression of CAT enzyme is sufficient to overcome the negative selection pressure provided by exposure to Chloramphenicol thereby facilitating bacterial outgrowth. The level of mCherry fluorescence similarly increased across all wells over the 3-day period indicating that the HCEC 1CT Cat-mCh cell monolayer continued to produce fluorescent protein in all wells regardless of bacterial outgrowth (S4A Fig).

However, in the presence of a mixed monolayer of DLD-1 Cma-ZsG1 and HCEC 1CT Cat-mCh cells, none of the wells show discolouration of culture media or outgrowth of bacterial colonies (Fig 5A (rows D-H) and 5B). Therefore, this is consistent with DLD-1 Cma-ZsG1 cells providing sufficient negative selection pressure to repress the growth of DH5α *E*. *coli* through the colicinogenic activity of secreted Colicin M, and preventing bacterial outgrowth despite the positive selection pressure supplied by the HCEC 1CT Cat-mCh cells which provide relief from the action of Chloramphenicol through expression of CAT. In support of this, the level of mCherry and ZsGreen1 fluorescence similarly increased in all wells over 3 days, indicating that both human cell lines in the mixed monolayer continued to produce fluorescent protein (S4 Fig). Moreover, the level of mCherry fluorescence is only slightly reduced when the cells are grown in a mixed monolayer with DLD-1 Cma-ZsG1 compared to HCEC 1CT Cat-mCh cells alone (S4B Fig). This suggests that the growth of HCEC 1CT Cat-mCh cells in the mixed monolayer is not greatly affected by the presence of DLD-1 Cma-ZsG1 cells, and so a reduced capacity to detoxify Chloramphenicol probably does not account for the absence of bacterial outgrowth in these wells.

None of the wells in which the mixed monolayer of DLD-1 Cma-ZsG1 and HCEC 1CT Cat-mCh cells was present showed bacterial outgrowth suggesting that DH5α *E*. *coli* do not intrinsically possess, and were unable to adapt to the imposed selection conditions and acquire, the characteristics required to overcome the combination of selection pressures applied during the 3 days of the experiment.

## Discussion

This study examines the potential for using a combination of positive and negative selection pressure–as designed in the Pioneer platform–to evolve (or discover) novel anti-cancer compounds produced by bacteria that have limited toxicity against healthy human cells. As proof of principle and to develop this platform, a transformed human cell line, namely the colorectal adenocarcinoma cell line DLD-1, was engineered to exert a negative selective pressure against *E*. *coli*. Through production and secretion of His-tagged Colicin M, these cells repress the growth of DH5α *E*. *coli* in liquid culture (Fig 2) and prevent outgrowth of these bacteria in semi-solid co-culture experiments (Fig 4A and 4C). These cells therefore exert negative selective pressure which would need to be overcome for the bacteria to survive. In contrast, the immortalised non-transformed human cell line HCEC 1CT, derived from colonic epithelial cells and representing healthy tissue in the context of a cancer patient, were engineered to express CAT. Through the activity of CAT these cells detoxify Chloramphenicol in conditioned media to promote the growth of DH5α *E*. *coli* in liquid culture (Fig 3) and enable bacterial outgrowth in semi-solid co-culture experiments (Fig 4B and 4D), thus promoting bacterial survival.

These engineered cell lines have been used to test a novel experimental screen (referred to as the Pioneer platform) in which both DLD-1 Cma-ZsG1 and HCEC 1CT Cat-mCh cells are established in a mixed monolayer and co-cultured with DH5α *E*. *coli* in semi-solid culture conditions supplemented with Chloramphenicol (Fig 1). Under these conditions, bacterial outgrowth is only possible in the presence of HCEC 1CT Cat-mCh cells which are required to detoxify Chloramphenicol (Fig 5A (row C) and 5B). However, the additional negative selective pressure provided by co-culture with DLD-1 Cma-ZsG1 cells prevents bacterial outgrowth under these conditions (Fig 5A (rows D-H) and 5B). Therefore, to cause outgrowth, DH5α *E*. *coli* must adapt to the imposed combination of selection conditions. This could hypothetically be achieved by a number of means: a) developing resistance to Colicin M; b) developing resistance to Chloramphenicol and general cytotoxic activity against both human cell types; c) developing selective cytotoxic activity against DLD-1 Cma-ZsG1 cells without affecting CAT production from HCEC 1CT Cat-mCh cells. Each of these possible events could be distinguished by monitoring the expression levels of fluorescent ZsGreen1 and mCherry proteins which label each of the human cell populations. However, none of these outcomes were observed using this experimental set-up and the DH5α *E*. *coli* did not adapt to overcome the selection conditions within 3 days and cause bacterial outgrowth.

Adaptation to the imposed selection conditions within 3 days is unlikely due to the limited number of generations. However, previous studies on the dynamics of adaptation in *E*. *coli* under selective pressure have shown adaptive mutations can be acquired within 11 days [23]. This is consistent with other microbial evolution studies whereby evolution of new traits typically requires <100 generations [24]. Therefore, extended incubation times to allow adaptation may yield a greater chance of bacterial outgrowth. However, in this experimental set-up the incubation time is limited by the requirement to sustain the growth and protein production of the human cell monolayer which diminishes at high cell confluency. However, at low cell confluency insufficient Colicin M and CAT protein is produced by the human cell monolayer to exert significant selection pressure, and it is not clear whether suboptimal strength of selection pressure may have contributed to the lack of bacterial adaptation and outgrowth in the Pioneer platform. Nevertheless, further optimisation of cell confluency, exogenous protein production and growth conditions could potentially enable the incubation period to be extended and the strength of the selection pressures to be modulated. Previous studies have also utilised BSCC platforms to enable longer term growth of bacterial species (including *E*. *coli*) in tumour spheroids derived from human cancer cells with stable colonisation for up to 2 weeks [7]. The incorporation of such techniques into the Pioneer platform could therefore potentiate a solution. Alternatively, surviving bacteria could be isolated from wells at the experimental endpoint and re-seeded into the Pioneer platform for multiple rounds of selection, analogous to the adaptive laboratory evolution experiments commonly used in microbial systems [24]. Other means to improve the probability of bacterial adaption could be to use hypermutator strains of *E*. *coli* or *E*. *coli* samples in which mutagenic variation has been induced through chemical or ultra-violet radiation treatment [25]. It could also be interesting to compare the abilities of these strains of *E*. *coli* to acquire resistance to purified Colicin M or Chloramphenicol as an indicator of their adaptive capacity to these selection pressures.

Natural bacterially-derived proteins and secondary metabolites and their derivatives have been a rich source of anti-cancer agents in the pharmacological arena, and there is a constant need to improve the efficacy and minimise off-target toxicity of these treatments, as well as discovering new agents with novel pharmacological activities to treat cancer and other diseases [4]. The Pioneer platform could prove a useful tool in this endeavour, providing an unbiased means to identify bacterial variants with novel contact-independent anti-cancer activities. This approach could serve as an alternative, or could possibly complement, rational drug design. Accordingly, it is possible that strains of *E*. *coli* with natural or engineered anti-cancer activity could be used and, through spontaneous adaptation, the Pioneer platform could select for optimised variants with superior anti-cancer activity and minimal off-target toxicity [4]. Indeed, this study would have benefited from a positive control strain of *E*. *coli* that exhibits contact-independent, selective anti-cancer cytotoxic activity to validate the Pioneer platform and demonstrate proof-of-principle. Reportedly, the bacteriocin Azurin preferentially penetrates human cancer cells where it exerts pro-apoptotic activities [26–29]. A recent study showed that a strain of *Salmonella typhimurium* equipped with Azurin as a therapeutic payload could reduce the size of tumour spheroids in BSCC experiments [7]. Therefore, a strain of *E*. *coli* engineered to secrete Azurin could be a promising candidate, but efforts to identify or engineer such a strain remain ongoing.

The experimental set-up of the Pioneer platform is compatible with high-throughput applications using discolouration of culture media as a surrogate read-out for bacterial outgrowth which could be quantified by the A525/425 ratio, while the fluorescence of ZsGreen1 and mCherry can be used as surrogate markers for the growth dynamics of the human cell populations using standard plate-readers. Although initially intended as a platform for the discovery of selective anti-cancer cytotoxic compounds produced as a result of spontaneous bacterial adaptation, the Pioneer platform described in this study could also be used to screen *E*. *coli* libraries for contact-independent cytotoxic activities against different human cell lines. These libraries could be derived from natural *E*. *coli* isolates or even artificially generated libraries (such as for Crispr interference screens or cells expressing secondary metabolite biosynthetic gene clusters from other microbial or plant sources), and replica plating techniques would complement high throughput applications. It is also possible that other substitutes for Colicin M and CAT could be explored so that susceptible bacteria would not be limited to *E*. *coli* and could therefore provide a wider repertoire of potential compounds through which cytotoxic activity could be attributed or developed.

The Pioneer platform is also not limited to comparing selective bacterial cytotoxic activities against human cancer *versus* immortalised non-transformed cells, but could potentially be used to compare any combination of cells that can be engineered to express the Colicin M and CAT proteins required and grow under these experimental conditions, including cells from other tissues or species. In particular, the platform could easily be adapted to different cancer types other than colorectal adenocarcinoma. The selection of suitable non-transformed immortalised cell lines for different tissue types may be more limiting, and in some cases cancer cell lines from different cancer sub-types may be a viable alternative (e.g. microsatellite instability (MSI) and CpG island methylator phenotype (CIMP) subtypes of colon cancer cell lines [30]). Indeed, there may be issues with how well these cell lines represent cancer *in vivo* or the normal healthy tissues from which they are derived. Consequently, it would be important to test any compounds with potential selective anti-cancer activity with a panel of representative cell lines as well as suitable *in vivo* models for cancer-targeted activity and toxicological effects.

In conclusion, this study describes the set-up and feasibility of a novel platform for the discovery of *E*. *coli* strains with contact-independent selective cytotoxic activity against co-cultured human cells, with potential utility for drug discovery, but further work remains to fully validate the proposed system.

## Supporting information

S1 Table A list of DNA oligonucleotides used in this study. (XLSX)

S1 Fig Cell surface expression of ZsGreen1 in DLD-1 Cma-ZsG1 cells. Representative ZsGreen1 fluorescence *versus* forward scattering (FSC-A) dot plots of DLD-1 (A) and DLD-1 Cma-ZsG1 (B) cells analysed by flow cytometry.
(TIF)

S2 Fig Colony formation of DLD-1 and HCEC 1CT cells grown in soft agar. Formation of colonies from DLD-1 and HCEC 1CT cells grown in soft agar for 14 days. A representative image from independent replicative experiments is shown; *n* = 3.
(TIF)

S3 Fig Cell surface expression of mCherry in HCEC 1CT Cat-mCh cells. Representative mCherry fluorescence *versus* forward scattering (FSC-A) dot plots of HCEC 1CT (A) and HCEC 1CT Cat-mCh (B) cells analysed by flow cytometry.
(TIF)

S4 Fig Levels of ZsGreen1 and mCherry fluorescence increase over 3 days in the Pioneer platform. Levels of mCherry (A) and ZsGreen1 (B) fluorescence in the Pioneer platform over 3 days in wells of a 96-well plate in the absence of human cells (grey circles), or with a monolayer of DLD-1 Cma-ZsG1 cells (green squares) or HCEC 1CT cells (orange diamonds) or a mix of both (blue triangles). Error bars show standard deviations of technical replicates, representative of independent replicative experiments; *n* = 2.
(TIF)

S1 Raw images (PDF)
