# Supplementary figures and images for: The Pioneer platform: A novel approach for selection of selective anti-cancer cytotoxic activity in bacteria through co-culturing with engineered human cells
Source: PLoS One. 2023 Jun 6;18(6):e0286741. doi: 10.1371/journal.pone.0286741 (PMC10243623; doi:10.1371/journal.pone.0286741)

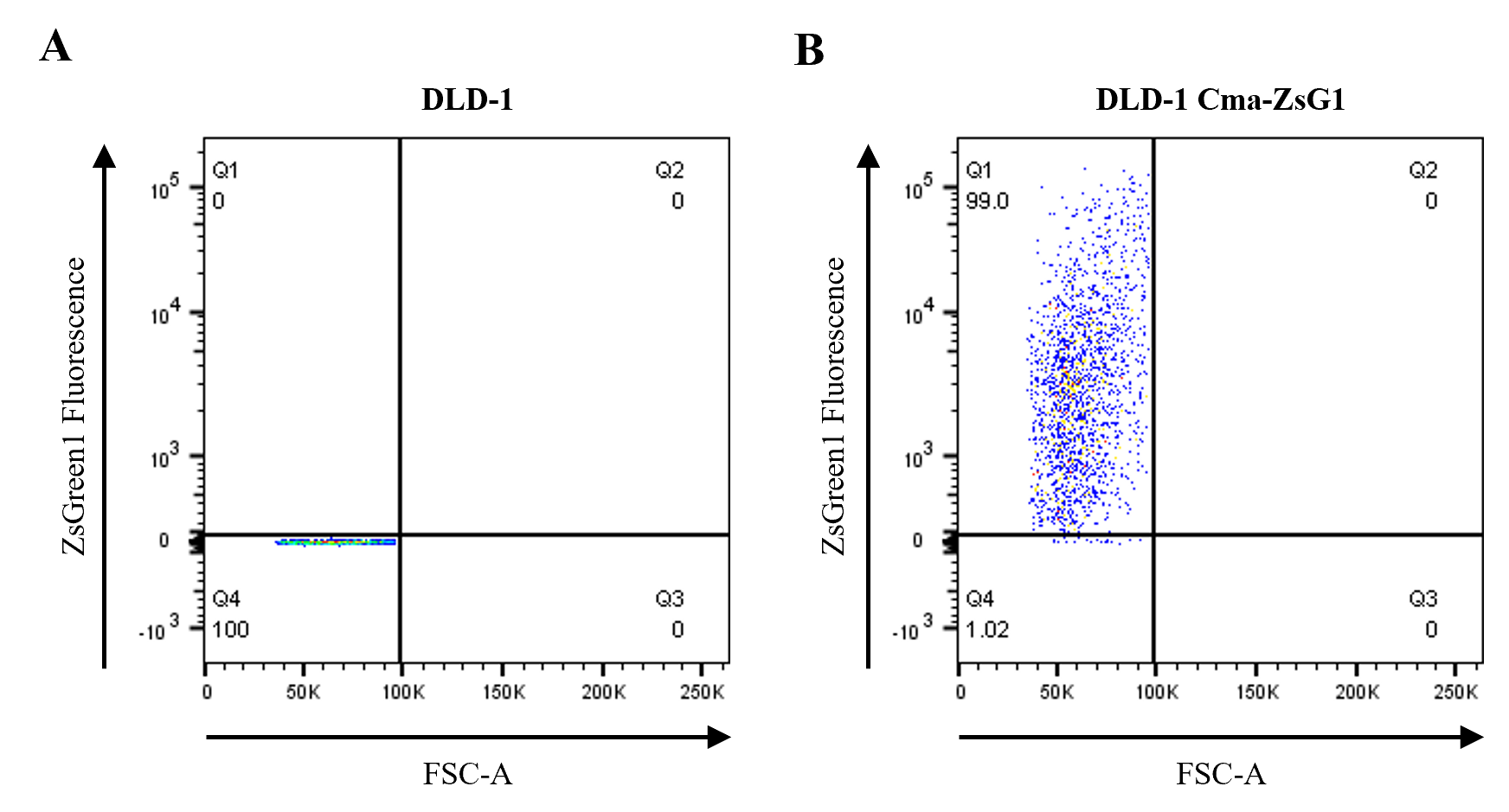

Supplement: S1 Fig — Representative ZsGreen1 fluorescence versus forward scattering (FSC-A) dot plots of DLD-1 (A) and DLD-1 Cma-ZsG1 (B) cells analysed by flow cytometry. (TIF) [file pone.0286741.s002.tif]

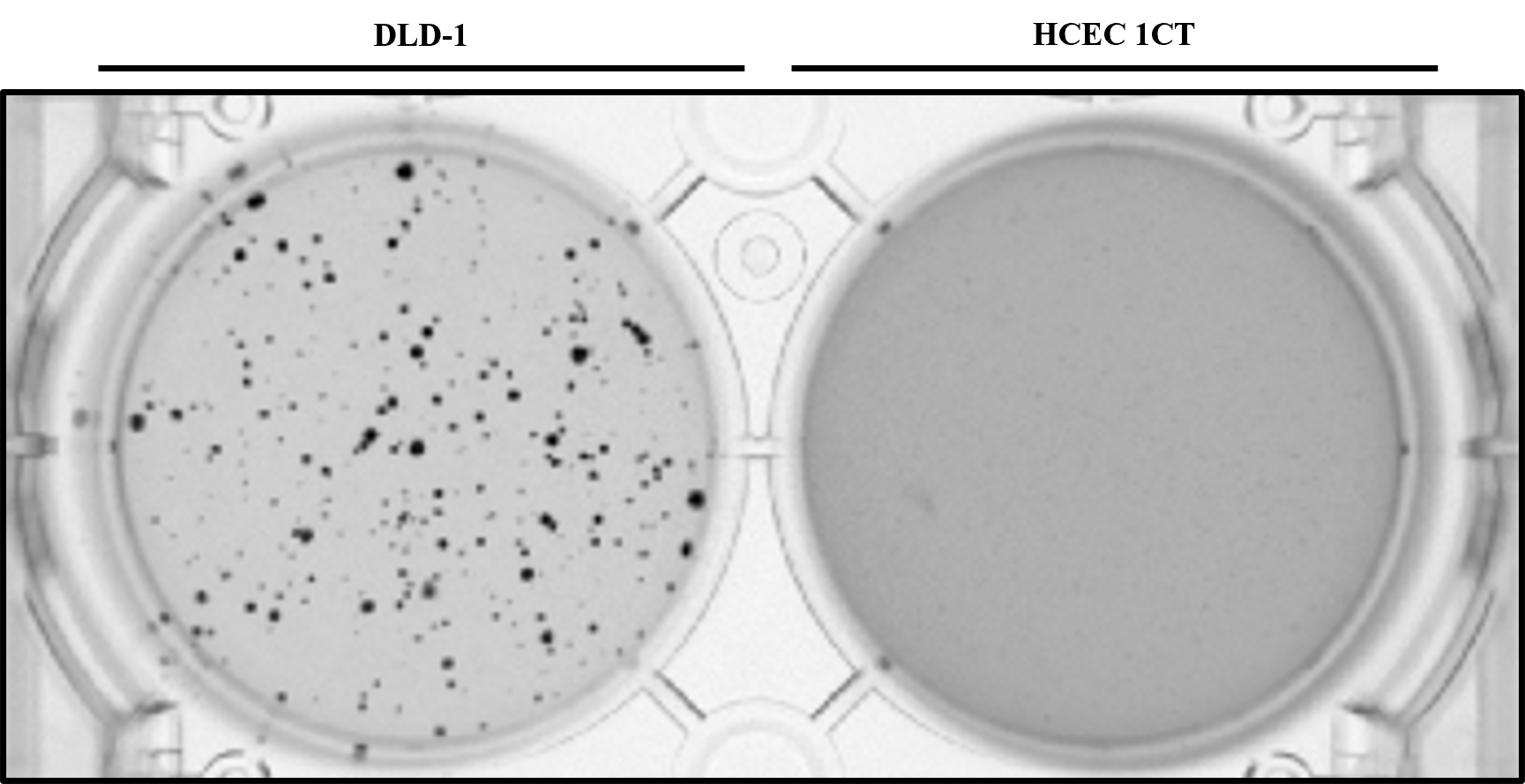

Supplement: S2 Fig — Formation of colonies from DLD-1 and HCEC 1CT cells grown in soft agar for 14 days. A representative image from independent replicative experiments is shown; n = 3. (TIF) [file pone.0286741.s003.tif]

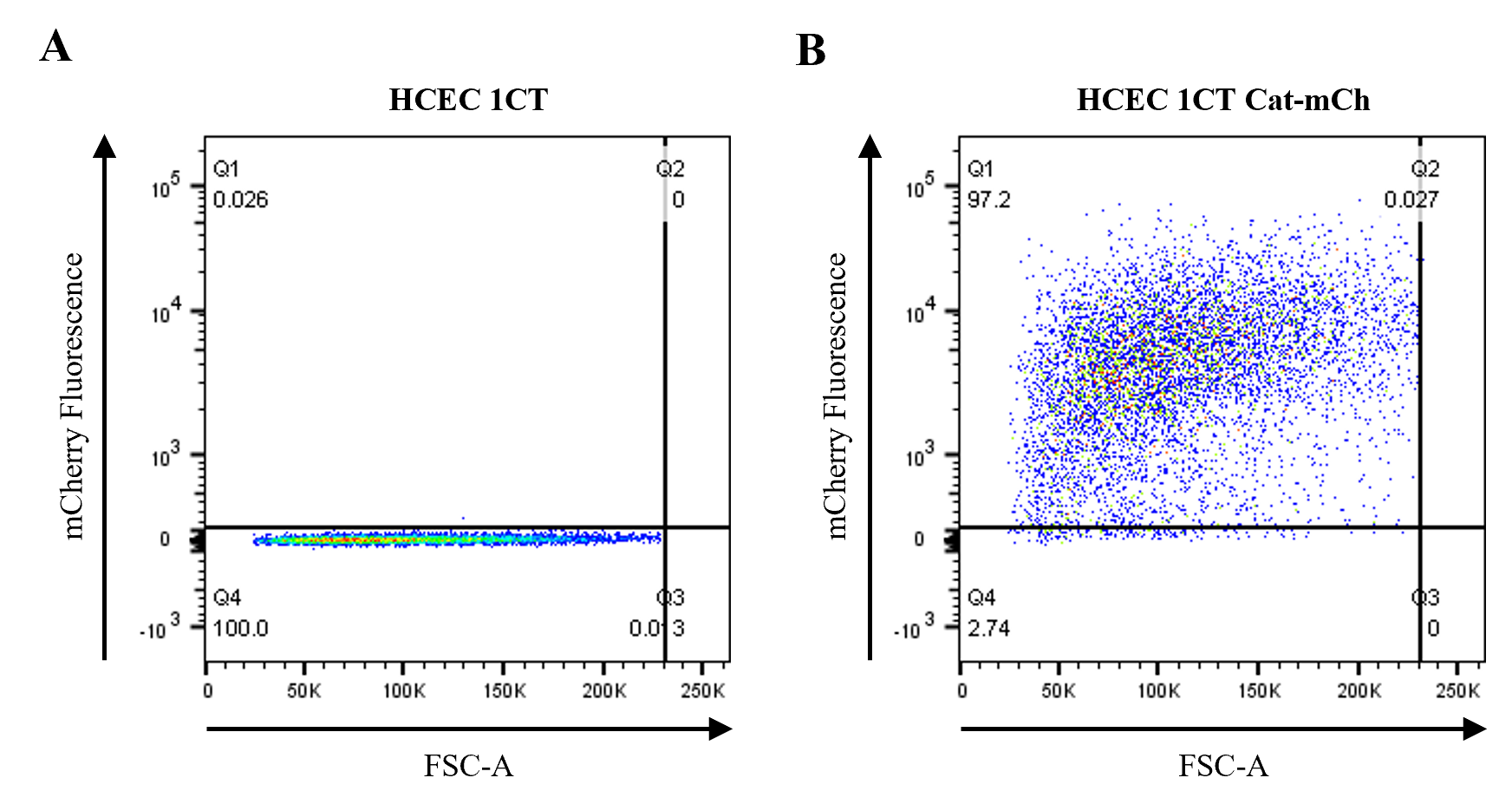

Supplement: S3 Fig — Representative mCherry fluorescence versus forward scattering (FSC-A) dot plots of HCEC 1CT (A) and HCEC 1CT Cat-mCh (B) cells analysed by flow cytometry. (TIF) [file pone.0286741.s004.tif]

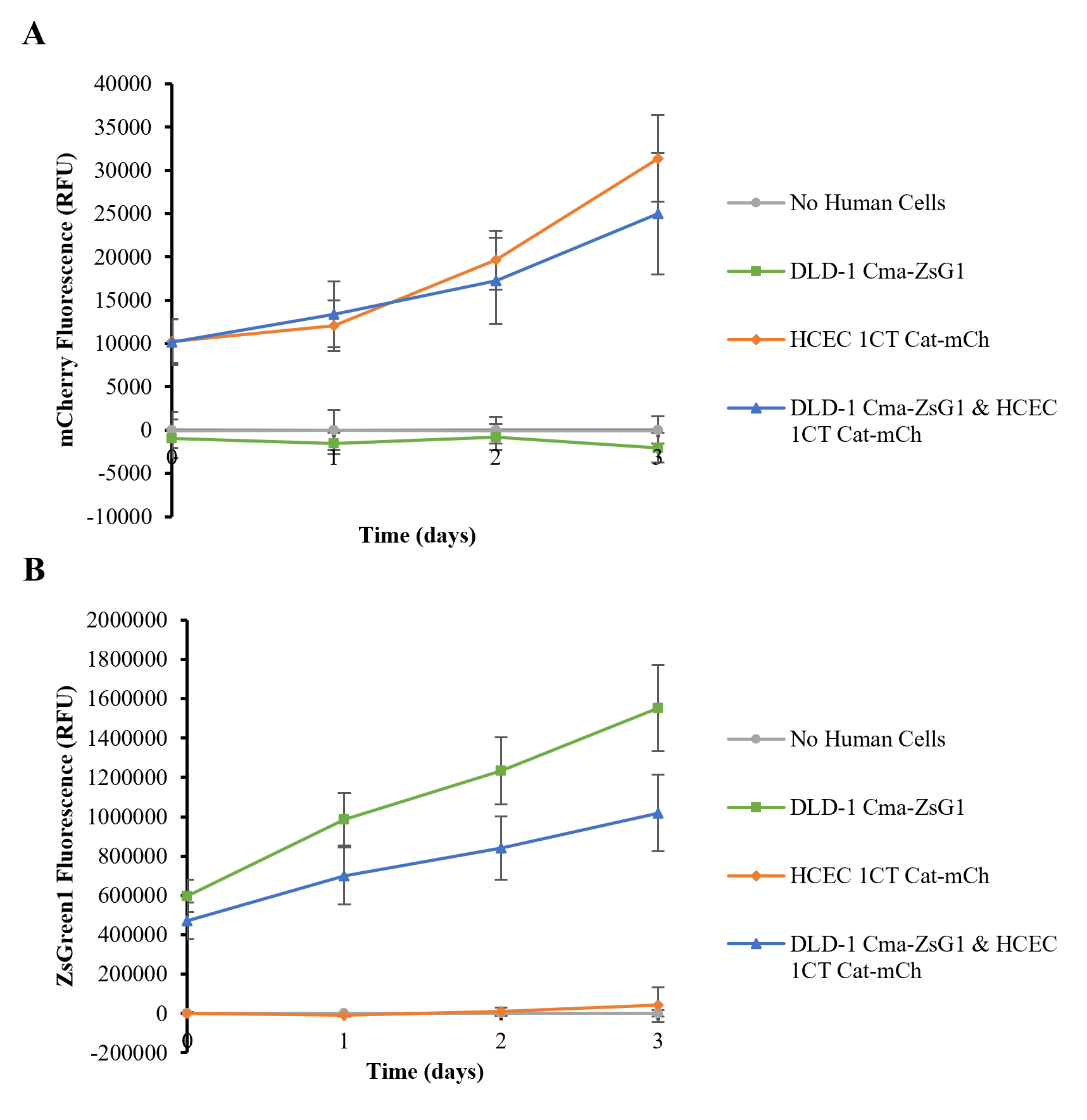

Supplement: S4 Fig — Levels of mCherry (A) and ZsGreen1 (B) fluorescence in the Pioneer platform over 3 days in wells of a 96-well plate in the absence of human cells (grey circles), or with a monolayer of DLD-1 Cma-ZsG1 cells (green squares) or HCEC 1CT cells (orange diamonds) or a mix of both (blue triangles). Error bars show standard deviations of technical replicates, representative of independent replicative experiments; n = 2. (TIF) [file pone.0286741.s005.tif]

Figure 2A

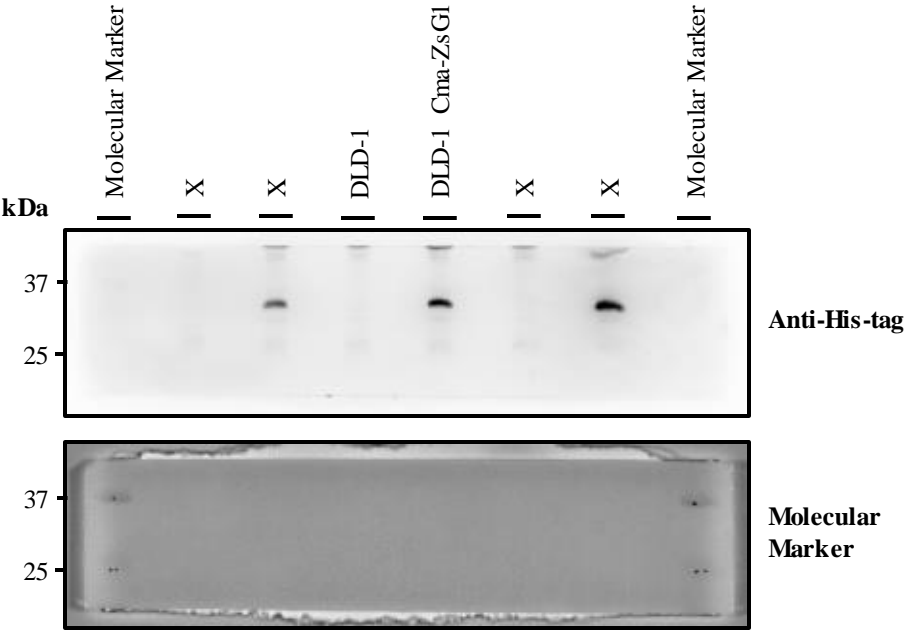

Figure 3A

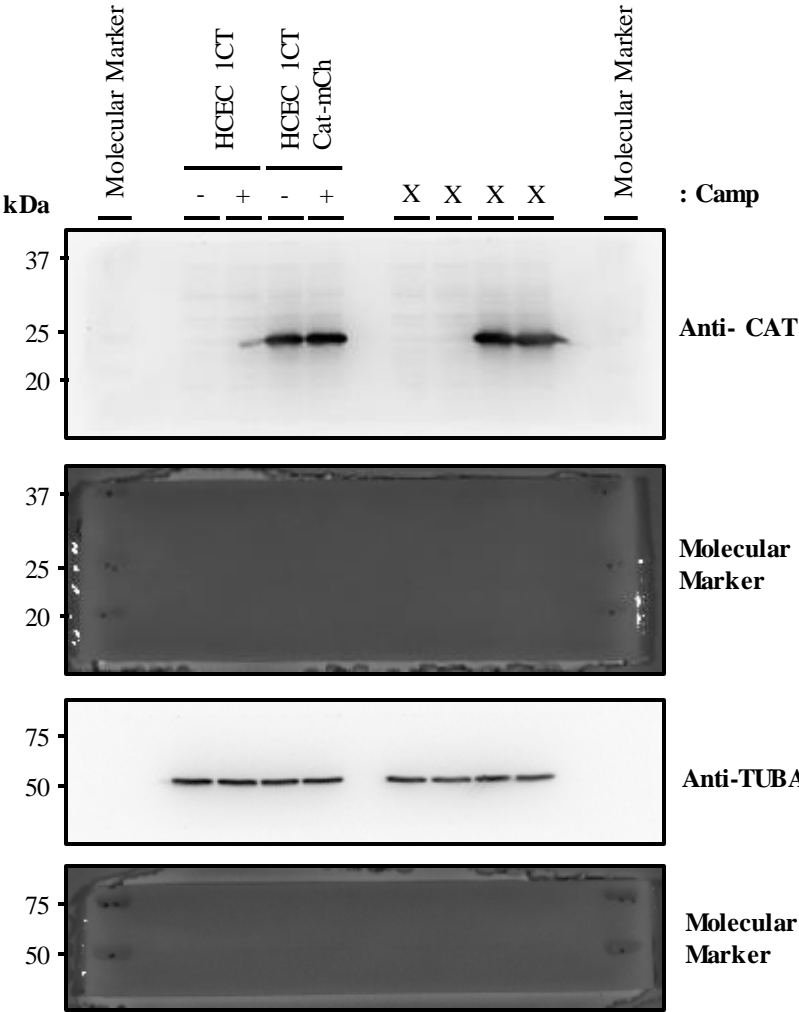

Supplement: S1 Raw images — (PDF) [file pone.0286741.s006.pdf]
